# Scabies: An Itchy Twitch

**DOI:** 10.7759/cureus.25120

**Published:** 2022-05-18

**Authors:** Hasan Shoaib, Collin J O'Bryan, Eliot Rapoport, Peter Phan

**Affiliations:** 1 Internal Medicine, University of Illinois College of Medicine Peoria, Peoria, USA; 2 Pulmonology, University of Illinois College of Medicine Peoria, Peoria, USA

**Keywords:** infectious dermatitis, maculopapular rash, developed world, north america, scabies

## Abstract

One of the most common and contagious infectious dermatological pathology is scabies. It is caused by tiny mites and spreads via skin-to-skin contact. It is most prevalent in children, the elderly, and the immunocompromised. Once diagnosed, the individual and all household contacts must be treated. Scabies has conventionally been considered a disease of the developing world and is prevalent in patients from a low socioeconomic status. Herein, we present an interesting case from a tertiary care hospital in North America.

## Introduction

Scabies is a common cause of infectious dermatitis in the developing world transmitted via skin contact by the mite *Sarcoptes scabiei* [1,2]. The World Health Organization reports that the global prevalence of scabies ranges from 0.2% to 70%. It can occur across all age groups; however, it is usually seen in children and adolescents [3]. Risk factors include inadequate hygiene, crowding, immunosuppression, and malnutrition. These factors are usually associated with people belonging to a low socioeconomic status, particularly in developing countries. It classically presents as pruritus, which is worse at night, with several potential cutaneous manifestations, including burrows, papules, nodules, and vesicles [4]. Burrows are pathognomonic for scabies. These lesions can be present in different regions of the body, most commonly on the wrists, palms, abdominal wall, and genitals [5]. In the last three decades, scabies has decreased globally; however, in North America, particularly in the higher socioeconomic demographic and elderly population, it has surprisingly increased [6]. The patient we discuss in this report belongs to a similar demographic.

## Case presentation

The patient was a 68-year-old male with a past medical history of paroxysmal atrial fibrillation, hypertension, and bipolar disorder. He also had a history of a diffuse maculopapular rash for the past several months. He presented to our facility after an episode of altered mental status with hypotension, hypothermia, septic shock, and a diffuse maculopapular rash.

**Figure 1:**
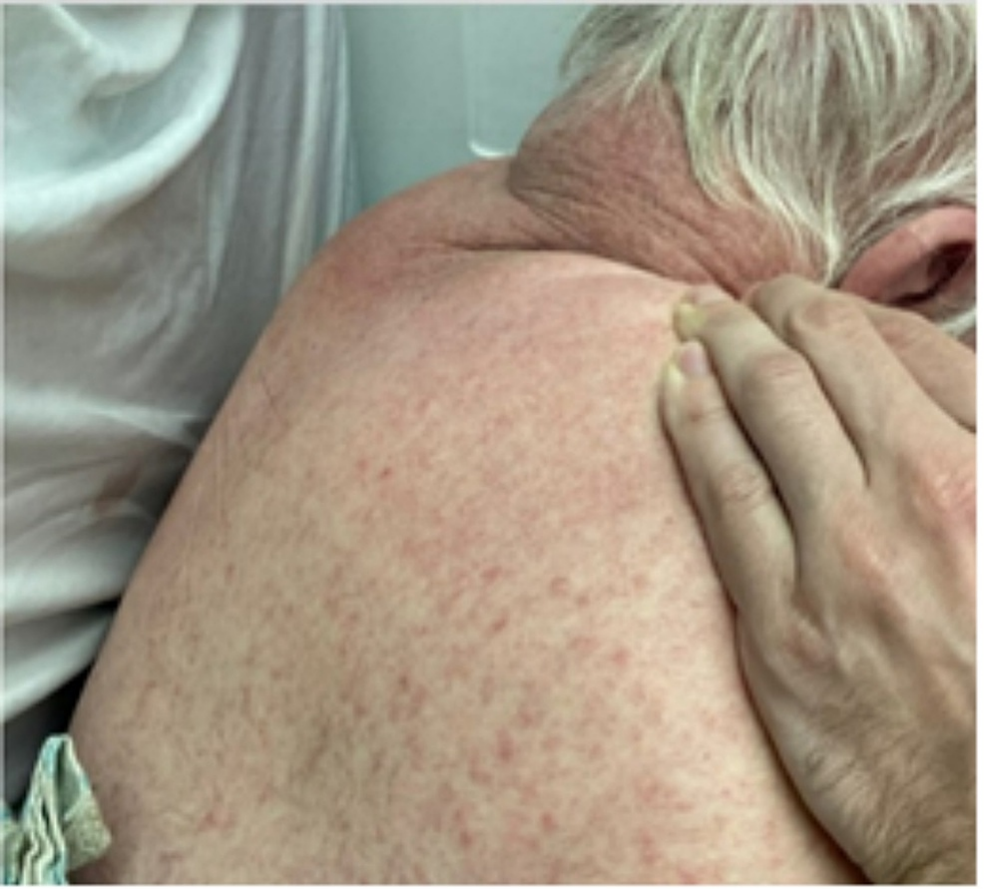
Diffuse maculopapular rash involving the upper back.

**Figure 2:**
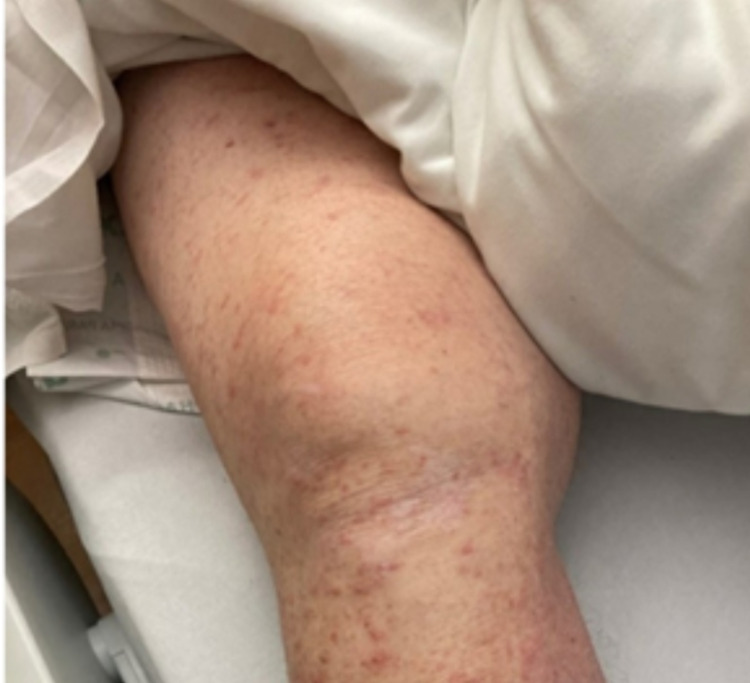
Diffuse maculopapular rash involving the right lower extremity with several excoriated papules.

The patient was diagnosed with acute adrenal insufficiency and sepsis. He was treated with glucocorticoids and vancomycin, with improvement in his clinical status; however, his rash remained. Upon further questioning, the patient reported intractable pruritus over the last several months that he and his roommate at the nursing home had both been experiencing. Physical examination revealed a diffuse maculopapular rash with widespread excoriations, burrows in the interdigital spaces, scattered xerosis, and poor hygiene, all suggestive of scabies dermatitis (Figures 1, 2). Upon evaluation of the lesions by a dermatologist, there was evidence of burrows in the interdigital spaces, scattered xerosis, and excoriation. The dermatoscope showed evidence of the delta-wing sign at the end of the burrows and scabies mites. A punch biopsy was also performed, which showed epidermal spongiosis with an underlying superficial perivascular infiltrate of lymphocytes with scattered eosinophils. It also revealed focal epidermal excoriation and ulceration, as well as organisms morphologically compatible with *Sarcoptes scabiei* in the epidermis. The patient was treated with 30 grams of topical permethrin once a week for two doses and oral ivermectin 0.2 milligrams per kilogram per dose that was also administered once a week for two doses with quick resolution of his rash and itching. The patient resided at a skilled nursing facility prior to his presentation. The facility was also notified to appropriately clean and treat as needed.

## Discussion

Our patient had several classic features of scabies. Scabies is transmitted by prolonged skin-to-skin contact [7]. Because of this mode of transmission, it is extremely common in settings that promote high levels of skin-to-skin contact, such as nursing homes, hospitals, schools, and homeless shelters [7]. Our patient was living in a nursing home when the rash started and reported that he and his roommate were both experiencing pruritus. He also had several features on physical examination that suggested the diagnosis including widespread excoriated papules, evidence of burrows in the interdigital spaces, scattered xerosis of the head, neck, trunk, and extremities, and poor hygiene. On dermoscopy, the mite head with a trailing burrow was visualized, which is commonly referred to as the “delta-wing jet” sign [7].

The patient also had some atypical features of scabies, most notably a diffuse maculopapular rash on his back. One study found only 3.9% of men and 10.5% of women with scabies had back involvement [8]. They proposed that because diffusion of scabies is via autoinoculation by scratching, the back may not be commonly involved due to the difficulty of reaching this area, although further studies are warranted [8]. The patient was also being treated with chronic corticosteroids for adrenal insufficiency, which can modify the clinical picture and delay diagnosis, a phenomenon referred to as “scabies incognito” [9]. In these patients, the lesions may become diffuse and appear as vesicles, pustules, or nodules [10]. It is also more common to develop crusted scabies in this patient population, which is a hyper infestation appearing as widespread thick keratotic crusts and scaly plaques [7].

Scabies is commonly misdiagnosed. In a single-center retrospective study in the United States, 45% of patients with scabies were previously misdiagnosed [7]. In our patient for example, the differential diagnosis included a drug rash and impetigo, among others. A high clinical suspicion for scabies must be maintained as it may mimic several dermatoses including atopic dermatitis, papular urticaria, dermatitis herpetiformis, prurigo nodularis, pityriasis rosea, and insect bites [9].

Given how common the misdiagnosis of scabies is, this case highlights typical and atypical findings to be aware of to prompt the clinical suspicion of scabies. Particularly in immunocompromised patients, such as our patient on chronic glucocorticoids, scabies may present with atypical features and an extremely widespread rash. With the proper diagnosis made, initiation of treatment can not only relieve the patient of symptoms but also prevent the spread to contacts, which is particularly important in settings such as hospitals and nursing homes as seen with our patient. Interestingly, while the global incidence of scabies decreases, there is an uptick of incidence in North America [6].

## Conclusions

Scabies conventionally has been recognized as a dermatological pathology of the developing world. We need to be wary that it can present in the developed world too, especially in patients with risk factors associated with this infectious dermatitis. It can sometimes present atypically as a diffuse rash and must be considered as a differential when appropriate.
